# Haemorrhagic mesenteric abscess in infective endocarditis: a unique cause of spontaneous haemoperitoneum

**DOI:** 10.1093/jscr/rjad023

**Published:** 2023-02-02

**Authors:** Phillipa Read, Patrick Walker, Tulsi Menon

**Affiliations:** Department of General Surgery, Royal Perth Hospital, Perth, WA 6000, Australia; Department of General Surgery, Royal Perth Hospital, Perth, WA 6000, Australia; Department of General Surgery, Royal Perth Hospital, Perth, WA 6000, Australia

## Abstract

Spontaneous haemoperitoneum (SH) is a rare but life-threatening condition with several well-recognized causes. SH often occurs in anticoagulated patients and requires urgent treatment to prevent mortality. SH is rarely associated with infective endocarditis (IE). To date, there are no reported cases of a haemorrhagic mesenteric abscess causing haemoperitoneum. We present the case of a warfarinized 30-year-old intravenous drug user with IE 2 weeks post-revision of a metallic valve who reported abdominal pain and subsequently suffered haemodynamic collapse. Abdominal computed tomography and damage-control laparotomy revealed haemoperitoneum secondary to an actively bleeding mesenteric nodule which was resected. Histopathology confirmed a haemorrhagic mesenteric abscess representing a rare sequela of IE and a novel cause of SH. Given that the incidence of IE appears to be increasing in people who inject drugs in Australia, the general surgeon should be aware of this clinical entity and the need for urgent intervention to prevent catastrophic sequelae.

## INTRODUCTION

Metastatic abscesses secondary to septic embolization from infective endocarditis (IE) has been well reported in the literature. However, very few cases of mesenteric abscesses secondary to IE have been reported. To our knowledge, no cases of haemorrhagic mesenteric abscesses causing haemoperitoneum have been published. This case highlights rare and potentially lethal sequelae of IE in a warfarinized patient.

## CASE PRESENTATION

**Figure 1:**
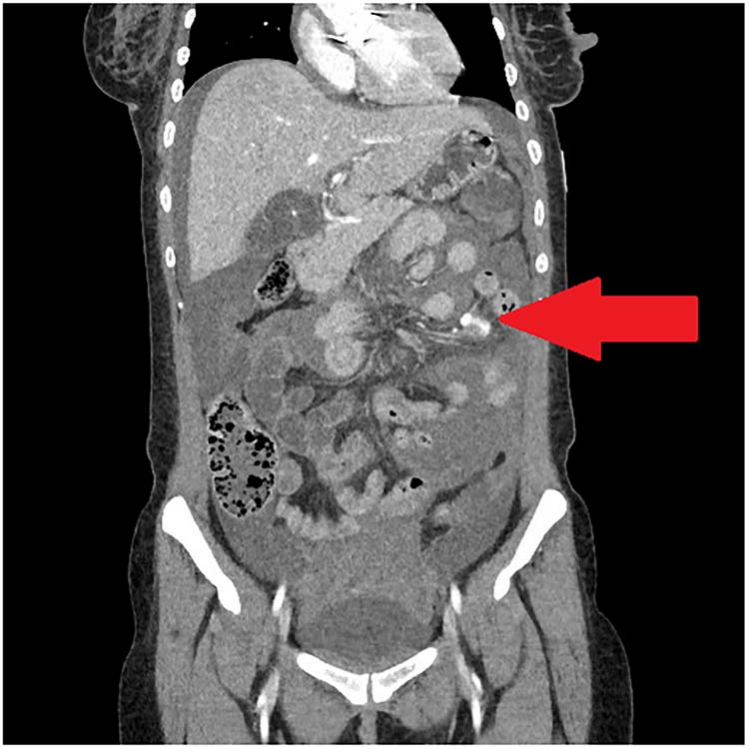
Portal-venous phase CT abdomen demonstrating haemoperitoneum and contrast extravasation in small bowel mesentery.

A 30-year-old woman was admitted with erratic behaviour and fever and found to have *Staphylococcus aureus* bacteraemia. She had a history of intravenous drug use and previous mechanical aortic valve replacement (AVR). Echocardiogram as part of her initial workup demonstrated aortic root abscess with evidence of septic emboli in the brain, spleen and bilateral kidneys. She underwent revision of AVR and warfarin was re-commenced. She remained an inpatient for ongoing intravenous antibiotics. After 2 weeks, she complained of severe abdominal pain, shortness of breath and dizziness. Over a period of hours, she became progressively tachycardic and hypotensive. A haemoglobin taken several hours after onset of pain was 76 g/L, representing a 25 g/L decrease from her baseline. WCC was elevated at 15 and INR was subtherapeutic at 2.2. Abdominal computed tomography (CT) with IV contrast demonstrated contrast material extravasating within the small bowel mesentery consistent with active bleeding (Fig. 1).

Shortly following the CT, she suffered haemodynamic collapse and required massive transfusion and damage-control laparotomy for control of haemorrhage. Upon entry of the abdomen, 2.5 L of blood and clot was evacuated and a 30 × 30 × 15 mm^3^ haemorrhagic nodule was identified with ongoing active bleeding. The lesion and associated 100 mm of dusky small bowel were resected with good haemostatic effect. 24 hours following damage-control laparotomy and normalization of physiological parameters, small bowel anastomosis and closure of the abdomen was performed. Heparin infusion was commenced 24 hours post-op. Her post-operative course was complicated by a superficial abdominal wall haematoma requiring operative washout following definitive abdominal wall closure. Recovery was otherwise uneventful, and she was re-commenced on warfarin day 9 post-evacuation of haematoma. Histopathology confirmed fat necrosis along with haemorrhage and abscess formation. There was adjacent reactive venulitis and thrombosis in the mesenteric veins. IV antibiotic therapy was continued until day 25 post-op, as directed by infectious disease physicians, and she was later discharged to the community.

## DISCUSSION

Spontaneous haemoperitoneum (SH) is defined as intra-abdominal bleeding in the absence of trauma [1]. It is a rare and life-threatening condition often caused by hepatic, splenic, vascular or gynecological pathology in anticoagulated patients [2]. In light of its rarity, SH is often unsuspected until time of imaging. SH requires immediate intervention and can be rapidly fatal. Clinical manifestations of SH include sudden onset abdominal pain, peritoneal signs, shock and acute anaemia. Diagnosis can be obtained with CT, which has been reported to have a sensitivity of 80% [3] or at time of laparotomy in cases of haemodynamic instability.

The incidence of IE in Australia is increasing, particularly among IV drug users [4]. IE can result in a wide variety of complications secondary to septic embolization, however, is infrequently associated with abdominal complications. Although there are cases of ruptured mycotic abdominal aneurysms in the setting of IE reported in the literature, there are no cases of a haemorrhagic mesenteric abscess reported as the cause of SH [5, 6].

Indeed, to our knowledge, there is only one published report of a mesenteric abscesses secondary to IE where the proposed pathogenesis was infarction of the mesentery, with subsequent bacterial proliferation and abscess formation [7]. Given that our patient had confirmed staphylococcal bacteraemia with multiple distant emboli and no alternative source of intra-abdominal infection, as well as concordant histopathological findings, our case appears to support this theory. We postulate that haemorrhagic transformation later occurred in the setting of anticoagulation leading to haemoperitoneum.

Haemorrhagic mesenteric abscess appears to be a unique cause of SH. Given that the incidence of IE appears to be increasing in people who inject drugs in Australia, the general surgeon should be aware of this clinical entity and the need for urgent intervention to prevent catastrophic sequelae. Our case highlights SH as an important differential in the warfarinized patient and introduces haemorrhagic mesenteric abscess as a unique cause of SH in patients with IE.
